# Structural characteristics of an insect group I chitinase, an enzyme indispensable to moulting

**DOI:** 10.1107/S1399004713033841

**Published:** 2014-03-19

**Authors:** Lei Chen, Tian Liu, Yong Zhou, Qi Chen, Xu Shen, Qing Yang

**Affiliations:** a School of Life Science and Biotechnology, Dalian University of Technology, 2 Linggong Road, Dalian, Liaoning 116024, People’s Republic of China; b State Key Laboratory for Biocontrol, Sun Yat-Sen University, Higher Education Mega Center, Guangzhou, Guangdong 510006, People’s Republic of China; c School of Software, Dalian University of Technology, 321 Tuqiang Street, Dalian, Liaoning 116620, People’s Republic of China; d State Key Laboratory of Drug Research, Shanghai Institute of Materia Medica, Chinese Academy of Sciences, 555 Zuchongzhi Road, Shanghai 201203, People’s Republic of China

**Keywords:** chitinase, glycosyl hydrolase, insects, *Ostrinia furnacalis*

## Abstract

Insects possess a greater number of chitinases than any other organisms. This work is the first report of unliganded and oligosaccharide-complexed crystal structures of the insect chitinase *Of*ChtI from *Ostrinia furnacalis*, which is essential to moulting. The obtained crystal structures were solved at resolutions between 1.7 and 2.2 Å. A structural comparison with other chitinases revealed that *Of*ChtI contains a long substrate-binding cleft similar to the bacterial chitinase *Sm*ChiB from *Serratia marcescens*. However, unlike the exo-acting *Sm*ChiB, which has a blocked and tunnel-like cleft, *Of*ChtI possesses an open and groove-like cleft. The complexed structure of the catalytic domain of *Of*ChtI (*Of*ChtI-CAD) with (GlcNAc)_2/3_ indicates that the reducing sugar at subsite −1 is in an energetically unfavoured ‘boat’ conformation, a state that possibly exists just before the completion of catalysis. Because *Of*ChtI is known to act from nonreducing ends, (GlcNAc)_3_ would be a hydrolysis product of (GlcNAc)_6_, suggesting that *Of*ChtI possesses an endo enzymatic activity. Furthermore, a hydrophobic plane composed of four surface-exposed aromatic residues is adjacent to the entrance to the substrate-binding cleft. Mutations of these residues greatly impair the chitin-binding activity, indicating that this hydrophobic plane endows *Of*ChtI-CAD with the ability to anchor chitin. This work reveals the unique structural characteristics of an insect chitinase.

## Abstract 2

The first crystal structure of an insect chitinase that is indispensable to moulting is revealed.

## 1. Introduction   

The glycosyl hydrolase family 18 (GH18) chitinases (EC 3.2.1.14) are enzymes that hydrolyze chitin, a β-1,4-linked *N*-­acetylglucosamine (GlcNAc) linear polymer, into chito­oligosaccharides. These enzymes are widely distributed in many organisms, including bacteria, fungi, insects, plants and mammals, and play roles in immunity and defence, digestion, pathogenicity and arthropod moulting (Arakane & Muthu­­krishnan, 2010 ▶). Understanding the structure–function relationship of these enzymes is essential for disease control and drug design.

The crystal structures of the catalytic domains (CADs) of free or ligand-complexed chitinases have been determined, including GH18 chitinases from bacteria (Perrakis *et al.*, 1994 ▶; van Aalten *et al.*, 2001 ▶; Papanikolau *et al.*, 2003 ▶; Songsiri­ritthigul *et al.*, 2008 ▶; Tsuji *et al.*, 2010 ▶; Hsieh *et al.*, 2010 ▶; Pantoom *et al.*, 2011 ▶; Payne *et al.*, 2012 ▶; Busby *et al.*, 2012 ▶; Malecki *et al.*, 2013 ▶), fungi (Hollis *et al.*, 2000 ▶; Rao *et al.*, 2005 ▶; Hurtado-Guerrero & van Aalten, 2007 ▶; Schüttelkopf *et al.*, 2010 ▶; Rush *et al.*, 2010 ▶; Yang *et al.*, 2010 ▶), plants (Cavada *et al.*, 2006 ▶; Ohnuma, Numata, Osawa, Mizuhara, Lampela *et al.*, 2011 ▶; Ohnuma, Numata, Osawa, Mizuhara, Vårum *et al.*, 2011 ▶) and mammals (Fusetti *et al.*, 2002 ▶; Olland *et al.*, 2009 ▶; Sutherland *et al.*, 2011 ▶), as well as GH19 chitinases from bacteria (Hoell *et al.*, 2006 ▶; Kezuka *et al.*, 2006 ▶) and plants (Song & Suh, 1996 ▶; Hahn *et al.*, 2000 ▶; Ubhayasekera *et al.*, 2007 ▶, 2009 ▶; Huet *et al.*, 2008 ▶; Ohnuma *et al.*, 2012 ▶) and a GH23 chitinase from a bacterium (Arimori *et al.*, 2013 ▶). The aforementioned structural information has shown that all GH18 chitinases employ a substrate-assisted retaining mechanism during catalysis, in which the C2-acetamido group of the substrate acts as the catalytic nucleophile (Tews *et al.*, 1997 ▶; Brameld & Goddard, 1998*a* ▶). However, the GH19 and GH23 family chitinases use an inverting mechanism (Brameld & Goddard, 1998*b* ▶; Arimori *et al.*, 2013 ▶).

Unfortunately, there is no structural information available for insect chitinases. Insects possess a greater number of chitinases than any other organisms; in fact, an insect may possess as many as eight groups of genes encoding GH18 chitinases, most of which are involved in chitinolytic processes (Zhu, Arakane, Banerjee *et al.*, 2008 ▶; Arakane & Muthu­krishnan, 2010 ▶). These chitinases differ in their domain compositions, enzymatic properties, expression patterns and tissue localizations. Although the crystal structure of the chitinase-like protein imaginal disc growth factor 2 (IDGF-2) from the fruit fly *Drosophila melanogaster* (*Dm*IDGF2) has been determined (Varela *et al.*, 2002 ▶), the reference value of this protein is low because *Dm*IDGF2 is not an active enzyme. *Dm*IDGF2 lacks the catalytic residues and has a blockage in the substrate-binding cleft (Varela *et al.*, 2002 ▶). For these reasons, *Dm*IDGF2 is referred to as a regulator rather than an enzyme.

Among the eight groups of insect chitinases, group I chitinases have been enzymatically well characterized and have been shown to function in a chitin-degradation process that is closely associated with insect moulting (Zhu, Arakane, Beeman *et al.*, 2008 ▶). When the transcription level of the group I chitinase *Tc*Cht5 from the red flour beetle *Tribolium castaneum* was downregulated during the larval, pharate pupal and pupal stages, the insects failed to shed their old cuticle and died during eclosion (Zhu, Arakane, Beeman *et al.*, 2008 ▶). Our previous work reported on the group I insect chitinase *Of*ChtI (previously called *Of*Cht5) from *Ostrinia furnacalis*, a species of moth that is the most destructive insect affecting corn production. *Of*ChtI is highly active in the hydrolysis of chitooligosaccharides, colloidal chitin and α-chitin, and exhibits kinetic properties that differ from those of bacterial and plant chitinases (Wu *et al.*, 2013 ▶). In this study, the catalytic domain of *Of*ChtI (*Of*ChtI-CAD) was crystallized both alone and complexed with oligosaccharides, providing the first structural insights into free or oligosaccharide-complexed insect chitinase. The structural investigations, together with site-directed mutagenesis studies, reveal that the insect chitinase *Of*ChtI possesses several unique structural features that distinguish this enzyme from other known chitinases.

## 2. Materials and methods   

### 2.1. Gene cloning and site-directed mutagenesis   

The gene fragment encoding *Of*ChtI-CAD (residues 19–407) was amplified from the full-length cDNA of *Of*ChtI (GenBank ID DQ294305) with the primers 5′-TGAAGC­TTACGTA**GAATTC**GCGGAGTCGGACAGCAGAGCG-3′ (forward) and 5′-CCGCCCTAGG**GAATTC**TTAATGATGATGATGATGATGAGAACGCGGTGGTGGAACAG-3′ (reverse). *Eco*RI restriction sites (bold) and a C-terminal 6×His affinity tag were introduced. The resulting PCR fragment was ligated into the pPIC9 vector (Invitrogen, Carlsbad, Califonia, USA) with an in-fusion HD cloning kit (Clontech, Palo Alto, California, USA).

The *Of*ChtI-CAD E148Q, E148A, F159A, F194A, W241A, Y290A, F194A/W241A, F159A/Y290A and F159A/F194A/W241A/Y290A mutants were produced using the QuikChange site-directed mutagenesis kit (Stratagene, La Jolla, California, USA), according to the manufacturer’s instructions. The mutated genes were sequenced to confirm that the desired mutations had been inserted.

### 2.2. Protein expression and purification   

The plasmid containing the *Of*ChtI-CAD gene was transformed into *Pichia pastoris* GS115 cells (Invitrogen, Carlsbad, California, USA) for the overexpression of recombinant *Of*ChtI-CAD. The cells were first grown in buffered glycerol complex medium (BMGY; 1% yeast extract, 2% peptone, 1% glycerol, 1.34% yeast nitrogen, 0.2% biotin, 100 m*M* potassium phosphate pH 6.0) at 303 K to an optical density (OD) of 2.0 at 600 nm. After reaching this OD, the cells were collected and resuspended in buffered methanol complex medium (BMMY; 1% yeast extract, 2% peptone, 1% methanol, 1.34% yeast nitrogen, 0.2% biotin, 100 m*M* potassium phosphate pH 6.0). Methanol [1%(*v*/*v*)] was added at 24 h intervals as an inducer. After 72 h of fermentation, the culture supernatant was harvested and subjected to ammonium sulfate precipitation (75% saturation) at 277 K. The precipitate was dissolved in buffer *A* (20 m*M* sodium phosphate, 0.5 *M* NaCl, 20 m*M* imidazole pH 7.4) and the sample was then loaded onto a HisTrap HP affinity column (5 ml; GE Healthcare, USA) pre-­equilibrated with buffer *A*. After washing the column, the target protein was eluted with buffer *B* (20 m*M* sodium phosphate, 0.5 *M* NaCl, 200 m*M* imidazole pH 7.4). The purity of the eluted protein was analyzed by SDS–PAGE and found to be >95%. The mutants were expressed and purified using the same procedure as used for wild-type *Of*ChtI-CAD.

### 2.3. Crystallization and data collection   

Crystallization screening of recombinant *Of*ChtI-CAD was performed using the commercially available Index, Crystal Screen and Crystal Screen 2 (Hampton Research, Riverside, California, USA) screens as well as The JCSG Core Suites I–­IV (Qiagen, Valencia, California, USA). Pure *Of*ChtI-CAD was spin-concentrated to 10 mg ml^−1^ in 50 m*M* HEPES pH 8.0, 100 m*M* NaCl. The hanging-drop vapour-diffusion crystallization experiments were set up at 277 K by mixing 1 µl *Of*ChtI-CAD and 1 µl reservoir solution. The protein crystallized after five months from a crystallization cocktail consisting of 100 m*M* HEPES pH 7.5, 25%(*w*/*v*) PEG 3350.

Crystals of the *Of*ChtI-CAD–(GlcNAc)_2/3_ complex were obtained by transferring native crystals to a stabilizing solution consisting of 5 m*M* (GlcNAc)_6_ (*N*′,*N*′′,*N*′′′,*N*′′′′,*N*′′′′′, *N*′′′′′′-hexaacetylchitohexaose; Santa Cruz Biotechnology Inc., Dallas, Texas, USA), 100 m*M* HEPES pH 7.5, 25%(*w*/*v*) PEG 3350. The crystals were soaked for approximately 30 or 60 min at room temperature. The E148A and E148Q mutants (at 10 mg ml^−1^) were co-crystallized with 5 m*M* (GlcNAc)_6_ in 100 m*M* HEPES pH 7.9, 23%(*w*/*v*) PEG 3350 and in 100 m*M* HEPES pH 8.0, 21%(*w*/*v*) PEG 3350, respectively.

These crystals were soaked for several minutes in reservoir solution containing 25%(*v*/*v*) glycerol as a cryoprotection agent and were subsequently flash-cooled in liquid nitrogen. Diffraction data were collected on BL-17U at the Shanghai Synchrotron Radiation Facility in China and the diffraction data were processed using the *HKL*-2000 package (Otwinowski & Minor, 1997 ▶).

### 2.4. Structure determination and refinement   

The structure of free *Of*ChtI-CAD was solved by molecular replacement with *Phaser* (McCoy *et al.*, 2007 ▶) using the structure of human acidic mammalian chitinase (PDB entry 3fxy; Olland *et al.*, 2009 ▶) as a model. The subsequent structures of the E148Q mutant and the complexes with oligosaccharides were solved using the coordinates of free *Of*ChtI-CAD as a model. The *PHENIX* suite of programs (Adams *et al.*, 2010 ▶) was used for structure refinement. The molecular models were manually built and extended using *Coot* (Emsley *et al.*, 2010 ▶). The stereochemistry of the models was checked by *PROCHECK* (Laskowski *et al.*, 1993 ▶). The coordinates of *Of*ChtI-CAD, *Of*ChtI-CAD with (GlcNAc)_2/3_, the E148A mutant with (GlcNAc)_2_ and the E148Q mutant were deposited in the Protein Data Bank as entries 3w4r, 3wl1, 3wl0 and 3wkz, respectively. All structural figures were generated using *PyMOL* (DeLano, 2002 ▶). The data-collection and structure-refinement statistics are summarized in Table 1 ▶.

### 2.5. Isothermal titration calorimetry   

Isothermal titration calorimetry (ITC) experiments were performed at 303 K using a MicroCal iTC_200_ System (MicroCal, Northampton, Massachusetts, USA). For the experiments with (GlcNAc)_3–6_, 0.2 m*M* E148Q mutant in 50 m*M* HEPES buffer pH 8.0 was placed in a reaction cell with a total volume of 202 µl, and 1–2 m*M* (GlcNAc)_3–6_ in the same buffer as the E148Q mutant protein was placed in the ITC syringe. Aliquots of 2–3 µl were injected into the reaction cell at 120 s intervals with a stirring speed of 1000 rev min^−1^. The titrations were typically completed after 13 or 20 injections. The background was measured by injecting (GlcNAc)_3–6_ into the buffer.

The ITC data were processed by the MicroCal *Origin* v.7.0 software that accompanied the ITC_200_ system. Prior to further data analysis, all data were corrected for the heat of dilution by subtracting the background. Using a nonlinear least-squares algorithm, the data were analyzed using the single-site binding model in the *Origin* software. Using this model, the stoichiometry (*n*), binding association constant (*K*_a_) and enthalpy change (Δ*H*) of the reaction were obtained. For (GlcNAc)_3_ binding, a two-site binding model was used. The reaction free-energy change (Δ*G*), the dissociation constant (*K*_d_) and the entropy change (Δ*S*) could be calculated from Δ*H* and *K*_a_ using Δ*G* = −*RT*ln*K*_a_ = *RT*ln*K*_d_ = Δ*H* − *T*Δ*S*, where *T* is the absolute temperature in kelvin and *R* represents the gas constant (1.98 cal K^−1^ mol^−1^).

### 2.6. Enzymatic assays and chitin-binding assays   

The enzymatic activities of wild-type *Of*ChtI-CAD and its mutants (F159A, F194A, W241A, Y290A, F194A/W241A, F159A/Y290A and F159A/F194A/W241A/Y290A) were determined using 4-nitrophenyl-β-chitobioside [*p*NP-(GlcNAc)_2_] and crystalline α-chitin as substrates. For *p*NP-(GlcNAc)_2_, the reaction mixture (60 µl) consisted of 20 n*M* enzyme and 0.1 m*M* *p*NP-(GlcNAc)_2_ in 50 m*M* sodium phosphate buffer pH 6.5. After incubation at 303 K for 10 min, 60 µl of 0.5 *M* sodium carbonate was added to the sample to stop the reaction. The amount of 4-nitrophenyl released was determined by measuring the absorbance at 405 nm. For crystalline α-chitin, the reaction mixture (100 µl) consisted of 40 n*M* enzyme and 1.0 mg ml^−1^ α-chitin in 50 m*M* sodium phosphate buffer pH 6.5. The reaction mixtures were incubated for 6 h at 303 K and then centrifuged at 13 000*g* for 5 min. The production of reducing sugars in the supernatant was followed with potassium ferriferrocyanide, and the levels of potassium ferriferrocyanide consumption, corresponding to the amount of reducing-sugar production, were quantified by measuring the absorbance at 420 nm (Imoto & Yagishita, 1971 ▶).

Binding assays were performed using crystalline α-chitin as the substrate. The reaction mixtures (500 µl) consisted of 1.0 mg ml^−1^ α-chitin and 0.3 mg ml^−1^ purified wild-type *Of*ChtI-CAD or its mutants in 50 m*M* sodium phosphate buffer pH 6.5 at 303 K with rotation at 250 rev min^−1^. At different time points, the samples were centrifuged at 13 000*g* for 5 min and the protein concentrations were determined from Bradford assays calibrated against bovine serum albumin (BSA).

## 3. Results   

### 3.1. Crystal structure of *Of*ChtI-CAD   

*Of*ChtI is a member of the insect group I chitinases, which are composed of an N-terminal signal region (residues 1–18), a CAD (residues 19–407), a PEST-like linker region (residues 408–491) and a C-terminal chitin-binding domain (residues 492–553). Our experiments showed that full-length recombinant *Of*ChtI was not stable and underwent autocleavage when incubated with the crystallization reagent, leaving a stable 43 kDa form with full catalytic activity towards the *p*NP-(GlcNAc)_2_ substrate. Thus, the truncated form of *Of*ChtI (*Of*ChtI-CAD; residues 19–407) was cloned, expressed and purified for crystallization.

*Of*ChtI-CAD crystals were obtained by vapour diffusion and the structure was determined by X-ray diffraction. The crystals belonged to space group *P*6_5_ and the structure was solved at a resolution of 1.7 Å. The crystal contained one molecule in the asymmetric unit. The final solved structure contained residues Arg24–Arg406 with two *N*-GlcNAc residues at the *N*-glycosylation sites Asn87 and Asn305.

**Figure 1:**
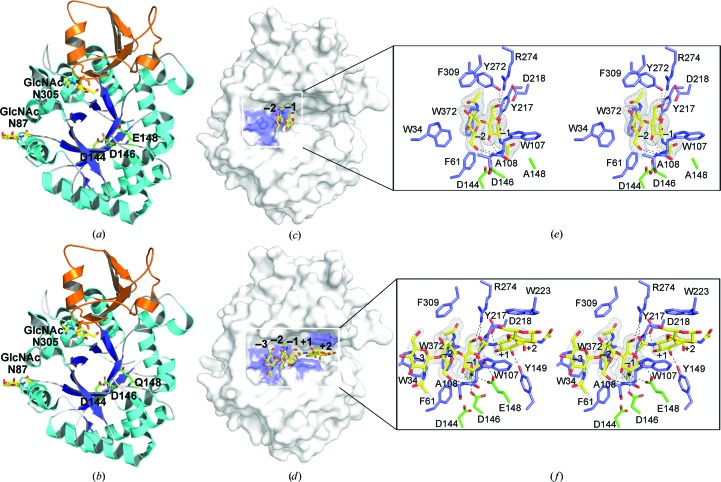
Overall structures of unliganded and oligosaccharide-complexed *Of*ChtI-CADs. (*a*, *b*) Cartoon representation of *Of*ChtI-CAD (*a*) and the E148Q mutant (*b*). The structure consists of two domains: a core domain with an (α/β)_8_ TIM-barrel fold (cyan, α-helices; blue, β-strands) and an insertion domain (orange). The catalytic residues and the *N*-GlcNAc residues at the *N*-glycosylation sites are shown as sticks with green and yellow C atoms, respectively. (*c*, *d*) Surface representations of E148A complexed with (GlcNAc)_2_ (*c*) and *Of*ChtI-CAD complexed with (GlcNAc)_2/3_ (*d*). The ligand is shown as a stick with yellow C atoms. The aromatic residues that stack with the sugar rings are shown in blue. The numbers indicate the subsite to which the sugar is bound. (*e*, *f*) Stereoview of the substrate-binding cleft with details of the interactions between (GlcNAc)_2_ and E148A (*e*) and between (GlcNAc)_2/3_ and *Of*ChtI-CAD (*f*). The ligand is represented as a stick with yellow C atoms and the 2*F*_o_ − *F*_c_ electron-density map around the ligand is contoured at the 1.0σ level. The catalytic residues and the amino acids that interact with the ligand are labelled and are shown as sticks with green and blue C atoms, respectively. The numbers indicate the subsite to which the sugar is bound. Hydrogen bonds are drawn as dashed lines.

Like most reported chitinases, *Of*ChtI-CAD folds into two distinct domains: a core domain and an insertion domain (CID). The core domain is composed of two separate regions (residues 24–271 and 350–406), forming a classical (β/α)_8_-barrel fold consisting of eight β-strands (β1–β8) at the centre tethered to eight α-helices (α1–α8). The CID (residues 272–349), which connects β7 and α7 of the core domain, is composed of five antiparallel β-strands flanked by two short α-­helices, forming one of the walls of the active pocket (Fig. 1 ▶ *a*). The chitinase signature motif ‘D*x*D*x*E’ (Asp144–Glu148) is located in the loop between strand β4 and helix α4 (Supplementary Fig. S1^1^). According to the catalytic mechanism (van Aalten *et al.*, 2001 ▶; Lu *et al.*, 2002 ▶; Synstad *et al.*, 2004 ▶; Vaaje-Kolstad *et al.*, 2004 ▶), the glutamate residue Glu148 is the catalytic acid/base, the aspartic acid residue Asp146 is critical for stabilizing the enzyme–substrate intermediate, and Asp144 appears to be crucial for keeping Asp146 protonated (Supplementary Fig. S2).

Based on sequence alignment with other GH18 chitinases, *Of*ChtI-CAD shares 40% sequence identity with *Hs*Cht from *Homo sapiens*, 30% with *Nt*ChiV from the plant *Nicotiana tabacum*, 30% with the fungal *Af*ChiB1 from *Aspergillus fumigatus* and 27% with the bacterial *Sm*ChiA and *Sm*ChiB from *Serratia marcescens*. The overall structure of *Of*ChtI-CAD is similar to these chitinases, with an r.m.s. deviation of 1.07 Å (349 C^α^ atoms) with *Hs*Cht (PDB entry 1guv; Fusetti *et al.*, 2002 ▶), 1.79 Å (317 C^α^ atoms) with *Nt*ChiV (PDB entry 3alf; Ohnuma *et al.*, 2011 ▶), 1.56 Å (325 C^α^ atoms) with *Af*ChiB1 (PDB entry 1w9p; Rao *et al.*, 2005 ▶), 1.53 Å (328 C^α^ atoms) with *Sm*ChiA (PDB entry 1ctn; Perrakis *et al.*, 1994 ▶) and 1.80 Å (328 C^α^ atoms) with *Sm*ChiB (PDB entry 1e15; van Aalten *et al.*, 2000 ▶) (Supplementary Table S1).

**Figure 2:**
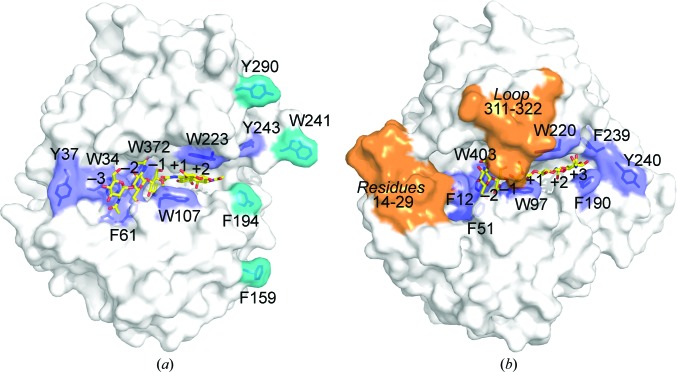
The substrate-binding clefts of the *Of*ChtI-CAD (*a*) and *Sm*ChiB (*b*) complexes. The carbohydrate-binding module and the linker of *Sm*ChiB are not shown. The (GlcNAc)_2/3_ bound to *Of*ChtI-CAD and the (GlcNAc)_5_ bound to *Sm*ChiB (PDB entry 1e6n; van Aalten *et al.*, 2001 ▶) are shown as sticks with yellow C atoms. The numbers indicate the subsites to which the sugar is bound. The aromatic residues in the substrate-binding clefts of *Of*ChtI-CAD and *Sm*ChiB are labelled and are shown as blue sticks. In *Of*ChtI-CAD, the four residues forming the hydrophobic plane are shown in cyan. In *Sm*ChiB, loop 311–322 forming the roof of the tunnel and residues 14–29 forming the blunted nonreducing end are labelled and shown in orange.

A long substrate-binding cleft was observed on the surface of *Of*ChtI-CAD and its volume was estimated to be 1628 Å^3^, with a surface area of 999 Å^2^, using the *CASTp* software (Dundas *et al.*, 2006 ▶). The cleft is composed of a set of aligned aromatic residues that includes Trp34, Tyr37, Phe61, Trp107, Phe194, Trp223, Trp241, Tyr243 and Trp372 (Fig. 2 ▶).

Surprisingly, a unique flat plane characterized by four aromatic residues, Phe159, Phe194, Trp241 and Tyr290, was observed on the surface of *Of*ChtI-CAD. These residues are solvent-exposed and located separately on the chain, but they come together to form a plane adjacent to the reducing end of the long substrate-binding cleft (Fig. 2 ▶ *a*; aromatic residues are highlighted in cyan). The possible function of these residues is discussed in §[Sec sec3.3]3.3.

### 3.2. Crystal structures of *Of*ChtI-CAD and its mutants with chitooligosaccharides   

To investigate the substrate-binding mode, three proteins, *Of*ChtI-CAD and its E148Q and E148A mutants (referred to in the following as E148Q and E148A, respectively), were crystallized by either co-crystallization or soaking with substrates. E148Q and E148A are variants with the catalytic residues mutated. Chitohexaose (GlcNAc)_6_, the longest chitooligosaccharide commercially available, was used as the substrate. The subsite nomenclature adopts the −*n* and +*n* nomenclature as utilized by Davies *et al.* (1997 ▶), where subsite −*n* represents the nonreducing end and +*n* represents the reducing end and cleavage occurs between the −1 and +1 subsites.

The crystal structure of E148Q at a resolution of 2.0 Å was obtained after a six-month incubation of E148Q with chito­hexaose. Unfortunately, no substrate was found in the structure. This indicates that E148Q possesses partial hydrolytic activity. Structural comparison of E148Q with wild-type *Of*ChtI-CAD indicates that the mutation does not introduce any changes in the catalytic active site or in the overall structure (Fig. 1 ▶ *b*).

Co-crystallization of E148A with (GlcNAc)_6_ was also a failure. Fortunately, the crystal structure of E148A complexed with (GlcNAc)_2_ was obtained at a resolution of 2.2 Å after six months. The structure is similar to that of wild-type *Of*ChtI-CAD, with an r.m.s. deviation of 0.16 Å based on the superimposition of 383 corresponding C^α^ atoms. The (GlcNAc)_2_ molecule binds at the −1 and −2 subsites (Fig. 1 ▶ *c*). The significant chitinase–substrate contacts are localized in subsites −1 and −2 as well as a number of amino-acid residues in *Of*ChtI-CAD that contribute to the binding of (GlcNAc)_2_. In the structure shown in Fig. 1 ▶(*e*), the O3 atom of the −1 sugar forms a hydrogen bond to the main-chain amide of Trp107. The pyranose ring of the −1 sugar stacks with the side chain of Trp372 *via* hydrophobic interaction. The O1 and O6 atoms form hydrogen bonds to the side chains of Tyr217 and Asp218/Tyr272/Arg274, respectively. The side chains of Trp34, Phe61 and Trp372 are involved in hydrophobic interactions with the sugar ring at the −2 subsite. The O6 atom of the −2 sugar forms a hydrogen bond to the main-chain amides of Trp107 and Ala108. A notable difference when compared with wild-type *Of*ChtI-CAD is that the side chain of Phe309 in E148A–(GlcNAc)_2_ rotates approximately 90° to stack with the acetamido group of the −2 sugar. A residue of this nature has not been observed previously in other chitinases from bacteria, humans or plants.

To obtain an enzyme–substrate complex, wild-type *Of*ChtI-CAD was first crystallized and then incubated with (GlcNAc)_6_ for various time intervals. We ultimately obtained a crystal structure at a resolution of 1.77 Å. Two sugars were complexed with the enzyme. The first was (GlcNAc)_3_, which occupied subsites −1, −2 and −3, and the other, to our surprise, was (GlcNAc)_2_, which localized between subsites +1 and +2 (Fig. 1 ▶ *d*).

The overall structure of *Of*ChtI-CAD complexed with (GlcNAc)_2/3_ is very similar to the unliganded enzyme, with an r.m.s. deviation for all matching atoms of 0.13 Å. It is important to note that the GlcNAc residue in (GlcNAc)_3_ at the −1 subsite is in an unfavoured ‘boat’ ^1,4^ *B* conformation (Fig. 1 ▶ *f*). Additionally, both the C2-acetamido group and O1 of this −1 sugar adopt different conformations from those in E148A–(GlcNAc)_2_. The O1 of the −1 sugar, which is the scissile glycosidic O atom, is within a distance of 2.5 Å of the catalytic O atom of the γ-carboxyl side chain of the catalytic proton donor Glu148. The C2-acetamido group of the −1 sugar is in a conformation that facilitates its O atom being 3.1 Å away from the C1, and this O atom forms a hydrogen bond to Tyr217. The C2-acetamido group of the −1 sugar is also stabilized by the side chains of the catalytic residues Glu148 and Asp146. The side chain of Asp146 rotates approximately 91° and then faces Glu148 (Fig. 1 ▶ *f*). This conformational change breaks the interaction between Asp146 and Asp144, but forms a hydrogen bond with the side chain of Glu148 instead. The sugar residue at the −2 subsite adopts the same conformation as observed in E148A–(GlcNAc)_2_. The interactions in this subsite are similar in the two complexed structures. The pyranose ring of the −3 sugar interacts with the aromatic residue Trp34 *via* a stacking interaction. For the interactions between (GlcNAc)_2_ and subsites +1 and +2, the O3 and O4 atoms of the +1 sugar form hydrogen bonds to residues Asp218 and Tyr149, respectively, the pyranose ring of the +1 sugar stacks with Trp107, and the pyranose ring of the +2 sugar stacks with Trp223 (Fig. 1 ▶ *f*).

### 3.3. Roles of the residues Phe159, Phe194, Trp241 and Tyr290   

**Figure 3:**
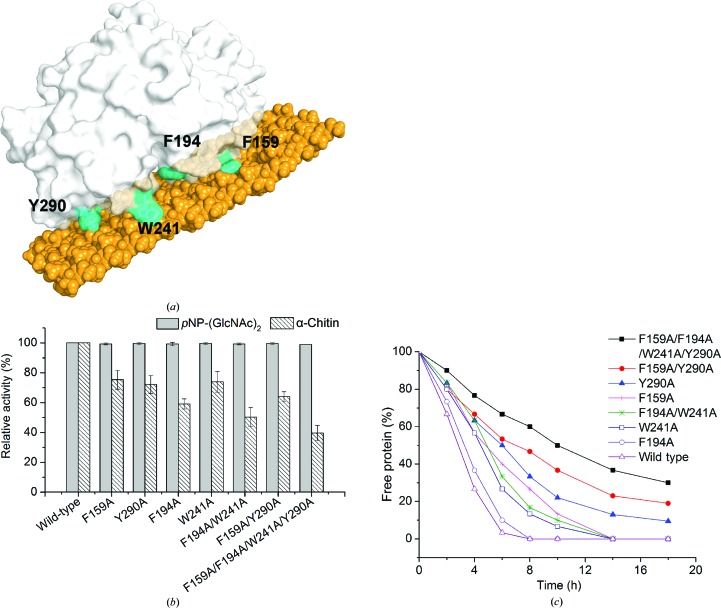
The effect of mutations of the four solvent-exposed aromatic residues on the hydrolytic activity and binding affinity. (*a*) The four aromatic residues, highlighted in cyan on the surface of *Of*ChtI-CAD, form a plane to anchor the plane of the crystalline chitin. A model of chitin is shown in orange. (*b*) The relative hydrolytic activities of the *Of*ChtI-CAD mutants for *p*NP-(GlcNAc)_2_ and crystalline α-chitin compared with wild-type *Of*ChtI-CAD. (*c*) The decrease in free protein concentration after binding of the wild-type and mutant *Of*ChtI-CADs to crystalline α-chitin was determined at different time points over 18 h.

The chitin-binding electrophoresis experiments indicated that *Of*ChtI-CAD has an affinity for crystalline α-chitin (Supplementary Fig. S3). The interaction between *Of*ChtI-CAD and α-chitin could not be destroyed by a high concentration of salt and acetic acid, suggesting that the affinity was strong (Supplementary Fig. S3). Based on the crystal structures, we suspect that a hydrophobic plane formed by the four solvent-exposed residues Phe159, Phe194, Trp241 and Tyr290 might contribute to the binding of *Of*ChtI-CAD to chitin (Fig. 3 ▶ *a*).

To confirm whether these surface aromatic residues participate in chitin binding, four single-residue mutants (F159A, F194A, W241A and Y290A), two double-residue mutants (F194A/W241A, F159A/Y290A) and one four-residue mutant (F159A/F194A/W241A/Y290A) were constructed. All of the *Of*ChtI-CAD variants were expressed and purified to homogeneity.

The enzymatic assays indicated that all of these mutants exhibit the same hydrolytic activities towards *p*NP-(GlcNAc)_2_, suggesting that the catalytic activity was not impaired when a short oligosaccharide was used as the substrate (Fig. 3 ▶ *b*). However, if the long crystalline α-chitin was used as the substrate, the mutations caused differing degrees of reduction in the enzymatic activity (Fig. 3 ▶ *b*). Phe194, which is the closest residue to the substrate-binding cleft, is the most crucial for chitin-hydrolysis activity. Mutation of any of the other three residues affected the chitin-hydrolysis activity to the same degree. Therefore, we deduce that Phe194 may be responsible for binding a single chitin chain during hydrolysis, while the other three residues are more likely to anchor the chitin plane.

The binding capacities of wild-type *Of*ChtI-CAD and its mutants were tested by incubating the proteins with α-chitin. The amount of protein bound to α-chitin was analyzed by determining the concentration of free protein in the supernatant at different time points. As shown in Fig. 3 ▶(*c*), the binding capacity decreased dramatically when the four residues were mutated. For the four-residue mutant, approximately 30% of the protein was retained in the supernatant even after an 18 h incubation. However, for the wild type, no free protein was left after a 6 h incubation. To determine the contribution of each residue, the binding ability of the four single-residue mutants was tested and concluded to be in the following order: Y290A < F159A < W241A < F194A. The mutation of Y290A and F159A caused a more drastic decrease than the mutation of W241A and F194A. The single-residue mutation of Y290A or F159A caused a more significant decrease than the double-residue F194A/W241A mutation, which indicated that residues Tyr290 and Phe159 are the major contributors to the binding ability. Mutation of Phe194 had very little effect on chitin binding, meaning that it was the smallest contributor.

The above results prove that the four residues forming a hydrophobic plane may not be involved in the catalytic process but instead in binding the chitin substrate.

### 3.4. Free-energy changes during the binding of *Of*ChtI to substrates   

**Figure 4:**
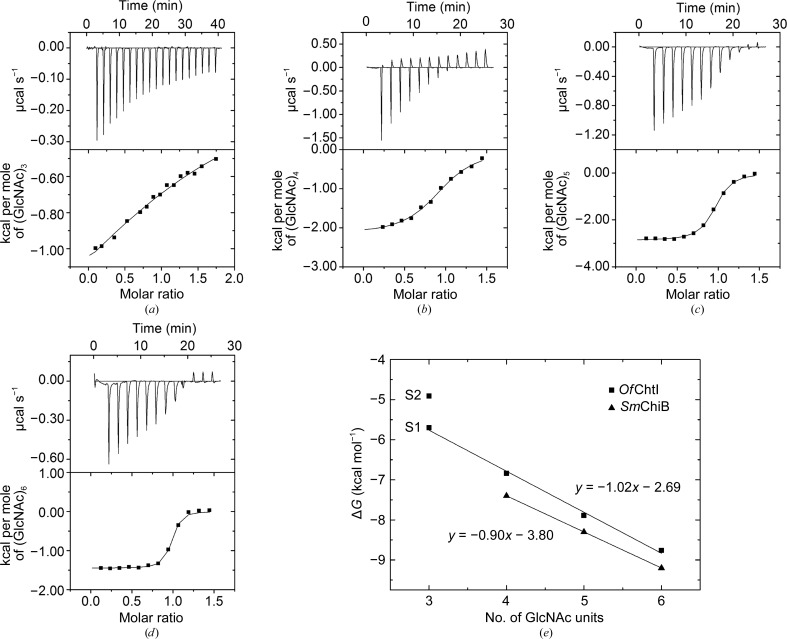
Thermograms and binding isotherms with theoretical fits for the binding of (GlcNAc)_3–6_ to the *Of*ChtI-CAD E148Q mutant: (*a*) (GlcNAc)_3_, (*b*) (GlcNAc)_4_, (*c*) (GlcNAc)_5_, (*d*) (GlcNAc)_6_. (*e*) Free-energy changes for (GlcNAc)_3–6_ binding to *Of*ChtI-CAD E148Q and (GlcNAc)_4–6_ binding to *Sm*ChiB E144Q (from Norberg *et al.*, 2010 ▶) relative to the number of GlcNAc units in the saccharide chain. (GlcNAc)_3_ binds to *Of*ChtI-CAD E148Q at two sites. S1 is the strong binding site with a low *K*_d_ value and S2 is the weak binding site with a high *K*_d_ value.

The substrate-binding energy changes for E148Q were measured by ITC analysis using (GlcNAc)_3–6_ as ligands. Because (GlcNAc)_2_ is the product of *Of*ChtI, (GlcNAc)_2_ was not included. The substrate binding was assessed in 50 m*M* HEPES pH 8.0 at 303 K. The thermograms and titration curves for (GlcNAc)_3–6_ are shown in Fig. 4 ▶. The derived thermodynamic parameters of binding, *K*_d_, Δ*H*, Δ*S* and Δ*G*, for (GlcNAc)_3–6_ are summarized in Table 2 ▶.

As shown in Fig. 4 ▶(*a*), the titration curve for (GlcNAc)_3_ binding to E148Q fit well to a two-site binding model with a stoichiometry of 1.84. (GlcNAc)_3_ bound to E148Q with an approximate fourfold affinity difference (*K*_d1_ = 76.9 µ*M* and *K*_d2_ = 284.1 µ*M*) in the two binding sites. For convenience, we named the site with the low *K*_d_ value the ‘strong binding site’ and the site with the high *K*_d_ value the ‘weak binding site’. Both types of binding were exothermic (Δ*H* < 0) and were entropically driven with an enthalpic contribution (Δ*H* < 0, |−*T*Δ*S*| > |Δ*H*|; Table 2 ▶).

For (GlcNAc)_4–6_, the titration curves fitted to a single-site binding model (Figs. 4 ▶ *b*, 4 ▶ *c* and 4 ▶ *d*). According to the *K*_d_ values of 11.3, 2.0 and 0.48 µ*M* for (GlcNAc)_4_, (GlcNAc)_5_ and (GlcNAc)_6_, respectively, the highest *K*_d_ value indicates that the binding of (GlcNAc)_4_ is the weakest, and an increase in the polymerization degree of GlcNAc by one unit results in a gain in affinity of approximately fivefold. The higher affinity for (GlcNAc)_6_ compared with (GlcNAc)_5_ suggests that there are at least six sugar-binding subsites in the substrate-binding groove, which is in accordance with the crystal structural data. The enthalpy values are in the range −1.45 to −2.89 kcal mol^−1^, indicating that the binding of (GlcNAc)_4–6_ is exothermic. Based on the entropy values (−*T*Δ*S*) of −4.67, −5.00 and −7.31 kcal mol^−1^ for (GlcNAc)_4_, (GlcNAc)_5_ and (GlcNAc)_6_, respectively, together with the fact that |−*T*Δ*S*| > |Δ*H*| (Table 2 ▶), we determined that the binding of all oligosaccharides examined is entropically driven. This evidence suggests that substrate binding is accompanied by desolvation and conformational changes of both the protein and its ligands.

To analyze the binding-energy changes of each ligand, the free energies of binding of (GlcNAc)_3–6_ were plotted against the polymerization degree of GlcNAc (Fig. 4 ▶ *e*). Notably, the binding energy of (GlcNAc)_3_ in the strong binding site, as well as the binding energies of (GlcNAc)_4_, (GlcNAc)_5_ and (GlcNAc)_6_, correlated well with the function *y* = −1.02*x* − 2.69 (where *y* is the free-energy change and *x* is the number of sugar residues, *i.e.* the polymerization degree). As shown in Fig. 4 ▶(*e*), an increase in *x*, namely an increase in the polymerization degree of GlcNAc [from (GlcNAc)_3_ to (GlcNAc)_6_], results in an average free-energy gain of approximately −1.0 kcal mol^−1^ per GlcNAc residue. Additionally, the binding of (GlcNAc)_6_ to *Of*ChtI releases 1.0 kcal mol^−1^ more free energy than the binding of (GlcNAc)_5_ to *Of*ChtI, demonstrating that *Of*ChtI possesses at least six substrate-binding subsites, as deduced from the crystal structures.

## 4. Discussion   

Here, we report the crystal structures of unliganded and oligosaccharide-complexed *Of*ChtI-CADs, which represent the first examples of insect GH18 chitinases.

### 4.1. Substrate binding sites   

Based on the ITC analysis of *Of*ChtI-CAD E148Q binding (GlcNAc)_4–6_, an increase in the polymerization degree of GlcNAc [from (GlcNAc)_3_ to (GlcNAc)_6_] results in an average free-energy gain of approximately −1.0 kcal mol^−1^ per GlcNAc residue. Similarly, according to Norberg *et al.* (2010 ▶), the binding of chitooligosaccharides to bacterial *Sm*ChiB E144Q showed that the free energies for binding (GlcNAc)_4_, (GlcNAc)_5_ and (GlcNAc)_6_ were −7.4, −8.3 and −9.2 kcal mol^−1^, respectively. The binding energies for (GlcNAc)_4_, (GlcNAc)_5_ and (GlcNAc)_6_ correlated well with the function *y* = −0.9*x* − 3.8, indicating that the free-energy gain for each added GlcNAc residue was −0.9 kcal mol^−1^ (Fig. 4 ▶ *e*). The above results demonstrate that the insect *Of*ChtI binds oligosaccharide substrates in similar manner as bacterial chitinase and that both enzymes possess at least six substrate-binding subsites.

### 4.2. *Of*ChtI-CAD possesses endo-acting activity   

Although most aromatic residues along the cleft are highly conserved, a detailed structural comparison between *Of*ChtI-CAD and the well characterized *Sm*ChiB indicates that these two enzymes possess different structural characteristics in the substrate-binding cleft. *Sm*ChiB has a tunnel-like cleft with a blunted nonreducing end. A unique loop (residues 311–322) forms the roof of the tunnel (Fig. 2 ▶ *b*), and the Asp316 residue in the loop directly interacts with Trp97 on the other side of the active pocket. The nonreducing end adjacent to subsite −3 is blocked by a short helix and a loop (residues 14–29). Moreover, the side chain of Phe12 at subsite −3 is positioned perpendicular to the sugar plane, making its interaction with the −3 sugar *via* a stacking interaction impossible (Fig. 2 ▶ *b*). In contrast, *Of*ChtI-CAD has a groove-like cleft with both the reducing end and the nonreducing end open. No short helices or loops are found at the ends of the cleft, and the cleft is exposed to solvent by the lack of the roof that is present in *Sm*ChiB (Fig. 2 ▶ *a*). The Trp34 residue at the deduced subsite −3 provides a strong stacking interaction with the pyranose ring of the −3 sugar (Fig. 2 ▶ *a*).

*Sm*ChiB is a well known exo-acting chitinase. The hypothesis is that an exo-chitinase, such as *Sm*ChiB, may be characterized by a tunnel-like and blunted substrate-binding cleft. Therefore, it follows that an endo-chitinase may lack these characteristics. For *Of*ChtI-CAD, the cleft is groove-like with both the nonreducing end and the reducing end open. The crystal structure of wild-type *Of*ChtI-CAD complexed with (GlcNAc)_2/3_ indicates that (GlcNAc)_3_ binds at subsites −1, −2 and −3, and the reducing sugar at subsite −1 sits in an energetically unfavoured ‘boat’ conformation. According to the deduced mechanism (Supplementary Fig. S2), this ‘boat’ conformation of the −1 sugar is likely to be a state that occurs just before the completion of catalysis, in which the product has left and the catalysis-associated Asp146 residue is still interacting with the −1 sugar. Because *Of*ChtI shows activity from the nonreducing ends of substrates (Wu *et al.*, 2013 ▶), the (GlcNAc)_3_ with a reducing sugar at subsite −1 suggests that (GlcNAc)_3_ could be a hydrolysis product of (GlcNAc)_6_. Thus, we provide direct evidence that a chitinase possessing a groove-like and open-ended cleft, such as *Of*ChtI-CAD, may possess endo-chitinase activity.

Taken together, although the GH18 chitinases employ the same catalytic mechanism and the substrate-binding cleft is constituted by a number of conserved aromatic residues, slight differences in the substrate-binding clefts could confer different cleavage modes of chitinases during chitinolytic processes.

### 4.3. Insect group I chitinase CAD possesses a surface hydrophobic plane   

Experimental results indicate that mutations of the four planar residues (Phe159, Phe194, Trp241 and Tyr290) resulted in a significant reduction in the hydrolysis of crystalline α-­chitin, but did not affect the hydrolysis of the short oligosaccharide substrate *p*NP-(GlcNAc)_2_. Moreover, the mutations seriously affected the binding affinity of α-chitin. Because the four residues are located far from the catalytic centre, we deduce that mutations causing a reduction in hydrolytic activity are related to the decrease in substrate-binding ability, which may be associated with a substrate-proximity effect. These results are the first to reveal the importance of surface-exposed aromatic residues of the catalytic domain during crystalline chitin hydrolysis.

According to a crystal structure-based alignment, the four aromatic residues are highly conserved in the insect group I chitinases from species including dipterans, lepidopterans, coleopterans, hemipterans and hymenopterans, but are absent in bacteria, fungi, plants and humans (Supplementary Fig. S1). This indicates that the four residues forming the hydrophobic plane to anchor the chitin are a conserved and unique characteristic of the group I insect chitinases.

## 5. Conclusions   

The first crystal structure of insect chitinase *Of*ChtI-CAD has now been described. The oligosaccharide-complexed crystal structure with the −1 reducing sugar sitting in a ‘boat’ conformation may provide evidence that a chitinase with a groove-like and open-ended cleft possesses endo-acting activity. A unique conserved hydrophobic plane in this insect chitinase increases the chitin-binding affinity. Because *Of*ChtI plays a vital role in insect moulting, this work provides a possibility for the development of new technologies for pest control and pest management.

## Supplementary Material

PDB reference: *Of*ChtI-CAD, 3w4r

PDB reference: complex with (GlcNAc)_2/3_, 3wl1

PDB reference: E148A mutant, complex with (GlcNAc)_2_, 3wl0

PDB reference: E148Q mutant, 3wkz

Supporting Information.. DOI: 10.1107/S1399004713033841/dw5087sup1.pdf

## Figures and Tables

**Table 1:** X-ray data-collection and structure-refinement statistics Values in parentheses are for the highest resolution shell.

	*Of*ChtI-CAD	E148Q	E148A–(GlcNAc)_2_	*Of*ChtI-CAD–(GlcNAc)_2/3_
PDB entry	3w4r	3wkz	3wlo	3wl1
Space group	*P*6_5_	*P*6_5_	*P*6_5_	*P*6_5_
Unit-cell parameters				
*a* = *b* (Å)	93.8	94.2	94.1	93.8
*c* (Å)	122.0	122.2	122.3	122.2
Wavelength (Å)	0.979228	0.978686	0.978686	0.978686
Temperature (K)	100	100	100	100
Resolution (Å)	50.0–1.70 (1.73–1.70)	50.0–2.00 (2.03–2.00)	50.0–2.20 (2.23–2.20)	50.0–1.77 (1.80–1.77)
Unique reflections	66488	41398	30971	58863
Observed reflections	1491430	702768	524755	952096
*R*_merge_	0.094 (0.361)	0.102 (0.367)	0.090 (0.368)	0.058 (0.424)
Average multiplicity	22.5 (22.5)	17.0 (17.0)	16.9 (17.0)	16.2 (15.0)
〈*I*/σ(*I*)〉	30.15 (12.80)	22.17 (10.77)	24.81 (12.32)	36.93 (8.55)
Completeness (%)	99.78 (99.65)	99.95 (99.83)	99.89 (98.93)	99.66 (99.05)
*R*/*R*_free_	0.1467/0.1594	0.1585/0.1856	0.1523/0.1777	0.1428/0.1686
Protein atoms	3086	3081	3077	3081
Water molecules	557	411	342	436
Other atoms	28	28	57	100
R.m.s. deviation from ideal				
Bond lengths (Å)	0.021	0.008	0.007	0.019
Bond angles (°)	1.790	1.160	1.100	1.760
Wilson *B* factor (Å^2^)	11.21	22.09	23.51	16.78
Average *B* factor (Å^2^)	15.70	21.00	22.20	22.00
*B* factor, protein atoms (Å^2^)	12.80	19.30	20.80	19.40
*B* factor, water molecules (Å^2^)	31.10	32.50	32.60	35.70
Ramachandran plot (%)				
Favoured	93.3	93.9	93.3	92.0
Allowed	6.7	6.1	6.7	8.0
Outliers	0.0	0.0	0.0	0.0

**Table 2:** Thermodynamic parameters of (GlcNAc)_3–6_ binding to the mutant E148Q derived from ITC

	*n*	*K*_d_ (µ*M*)	Δ*G* (kcal mol^−1^)	Δ*H* (kcal mol^−1^)	−*T*Δ*S* (kcal mol^−1^)
(GlcNAc)_3_	1.84 ± 0.12	*K*_d1_, 76.9 ± 18.9; *K*_d2_, 284.1 ± 97.4	Δ*G*_1_, −5.70; Δ*G*_2_, −4.91	Δ*H*_1_ −1.47 ± 0.15; Δ*H*_2_, −1.21 ± 0.34	−*T*Δ*S*_1_, −4.23; −*T*Δ*S*_2_, −3.70
(GlcNAc)_4_	0.93 ± 0.01	11.3 ± 1.5	−6.84	−2.17 ± 0.05	−4.67
(GlcNAc)_5_	0.92 ± 0.02	2.0 ± 0.2	−7.89	−2.89 ± 0.23	−5.00
(GlcNAc)_6_	0.93 ± 0.02	0.48 ± 0.09	−8.76	−1.45 ± 0.02	−7.31

## Footnotes

1 Supporting information has been deposited in the IUCr electronic archive (Reference: DW5087).
