# Supplementary material for: Structural characteristics of an insect group I chitinase, an enzyme indispensable to moulting
Source: Acta Crystallogr D Biol Crystallogr. 2014 Mar 19;70(Pt 4):932–42. doi: 10.1107/S1399004713033841 (PMC3975886; doi:10.1107/S1399004713033841)
Supplement: Supplementary file 1 [file d-70-00932-sup1.pdf]

Table S1 Sequence identities of *Of*ChtI-CAD with other structure-known chitinases from different species.

Species	PDB	Identity (%)	RMSD
Homo sapiens	3fxy	43	0.90
	1guv	40	1.07
Nicotiana tabacum	3alf	30	1.79
Bacillus circulans	1itx	29	1.07
Arabidopsis thaliana	3aqu	27	1.56
Serratia marcescens	1e15	27	1.80
	1ctn	27	1.53
Lactococcus lactis	3ian	27	2.74
Aspergillus fumigatus	1w9p	27	1.56
	2y8v	26	2.87
Vibrio harveyi	3b8s	26	1.26
Yersinia entomophaga	3oa5	26	1.80
Bacteroides thetaiotaomicron	3co4	25	2.20
Coccidioides immitis	1d2k	25	1.16
Arthrobacter tad20	1kfw	24	1.52
Clonostachys rosea	3g6l	23	1.25
Moritella marina	4hmc	22	2.67
Bacillus cereus	3n15	22	2.67
Crocus vernus	3sim	21	2.47

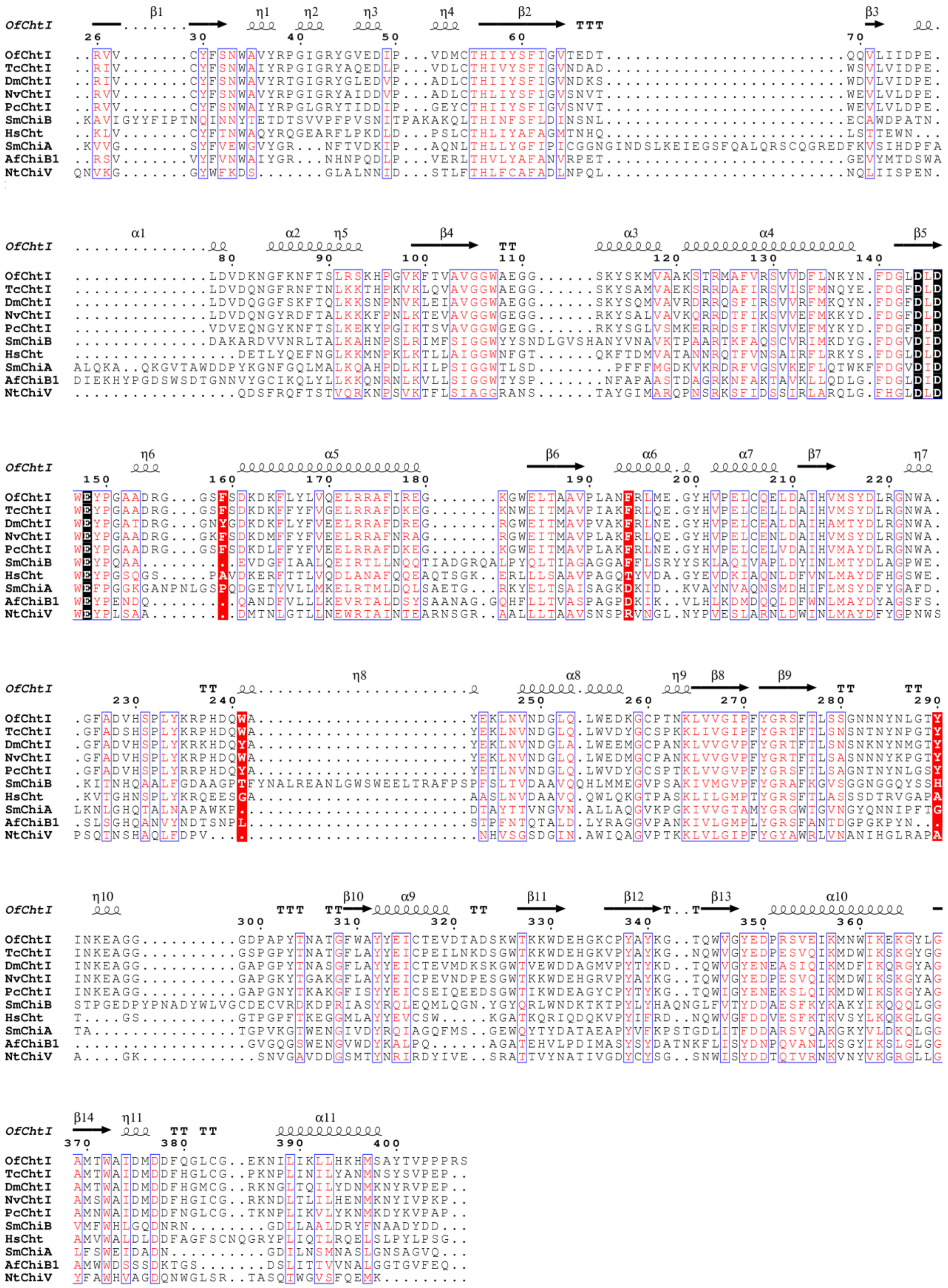

Figure S1

Structure-based sequence alignment of *Of*ChtI-CAD with other GH18 chitinases from various species. The insect Group I chitinases include *Of*ChtI from the lepidopteran insect *Ostrinia furnacalis* (Genbank ABB97081, PDB entry 3w4r), *Pc*ChtI from the hemipteran insect *Poophilus costalis* (Genbank AFW03959), *Nv*ChtI from the hymenopteran insect *Nasonia*

vitripennis (Genbank NP_001155084.1), *Tc*ChtI from the coleopteran insect *Tribolium castaneum* (Genbank AAV74190), and *Dm*ChtI from the dipteran insect *Drosophila melanogaster* (Genbank AAF54987). The other known chitinases include *Sm*ChiA (PDB entry 1ctn) and *Sm*ChiB (PDB entry 1e15) from *Serratia marcescens*, *Hs*Cht from *Homo sapiens* (PDB entry 1lg1), *Af*ChiB1 from *Aspergillus fumigatus* (PDB entry 1w9p), and *Nt*ChiV from *Nicotiana tabacum* (PDB entry 3alf). The secondary structure elements of *Of*ChtI-CAD are shown and labeled. The conserved catalytic residues are labeled with black background blocks. The four aromatic residues, which form a hydrophobic plane on the surface of *Of*ChtI-CAD, are highly conserved in the insect Group I chitinases but are absent in those from other species. They are labeled with red background blocks. The sequence alignment was generated by PROMALS3D (Pei *et al.*, 2008) and the picture was generated by ESPript (Gouet *et al.*, 2003).

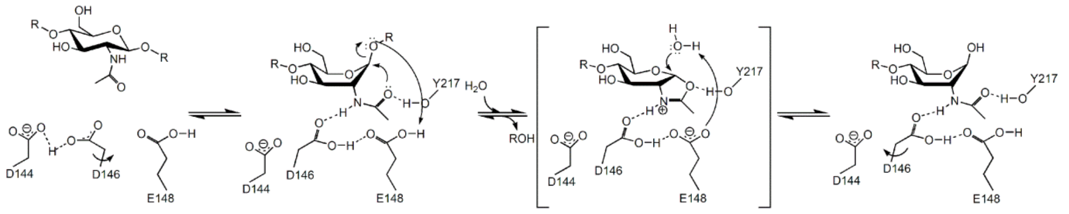

Figure S2

Proposed catalytic mechanism of family 18 chitinases, based on mechanism deduced by van Aalten *et al.* (2001).

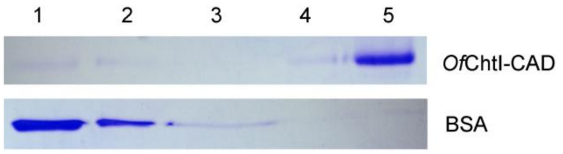

Figure S3

The binding of *Of*ChtI-CAD to crystalline α-chitin. The mixtures (50 µl) contained 10 µg *Of*ChtI-CAD and 1.0 mg crystalline α-chitin in 50 m*M* sodium phosphate buffer, pH 6.5. The mixtures were incubated at 303 K for 3 h and then centrifuged at 13 000*g* for 5 min. The supernatant was collected and the pellet was washed in 50 µl of 50 m*M* sodium phosphate buffer, pH 6.5, 50 m*M* sodium phosphate buffer, pH 6.5, containing 1*M* NaCl, and 0.1 *M* acetic

acid in turn. After centrifugation of each wash, the supernatants were collected. Finally, the pellet and all collected supernatants were subjected to 10% SDS-PAGE. Lane 1, unbound fraction; lane 2, wash fraction in 50 m*M* sodium phosphate; lane 3, wash fraction in 50 m*M* sodium phosphate containing 1*M* NaCl; lane 4, wash fraction in 0.1 *M* acetic acid; lane 5, bound fraction. BSA was used as a control.
